# Understanding the Associations between Executive Function and Psychological Variables in Fibromyalgia Syndrome: A Network Analysis Approach

**DOI:** 10.3390/healthcare12161678

**Published:** 2024-08-22

**Authors:** Juan C. Pacho-Hernández, Francisco G. Fernández-Palacios, Ángela Tejera-Alonso, César Fernández-de-las-Peñas, Umut Varol, Juan A. Valera-Calero, Luis M. Fernández-Méndez, Margarita Cigarán-Mendez

**Affiliations:** 1 Department of Psychology, Universidad Rey Juan Carlos, 28922 Alcorcón, Spain; juancarlos.pacho@urjc.es (J.C.P.-H.); angela.tejera@urjc.es (Á.T.-A.); margarita.cigaran@urjc.es (M.C.-M.); 2 Escuela Internacional de Doctorado, Universidad Rey Juan Carlos, 28933 Alcorcón, Spain; au.varol.2022@alumnos.urjc.es; 3 Department Physical Therapy, Occupational Therapy, Rehabilitation and Physical Medicine, University Rey Juan Carlos, 28922 Alcorcón, Spain; cesar.fernandez@urjc.es (C.F.-d.-l.-P.); luismifm_92@hotmail.com (L.M.F.-M.); 4 Department of Radiology, Rehabilitation and Physiotherapy, Faculty of Nursery, Physiotherapy and Podiatry, Complutense University of Madrid, 28040 Madrid, Spain; juavaler@ucm.es; 5 Grupo InPhysio, Instituto de Investigación Sanitaria del Hospital Clínico San Carlos (IdISSC), 28040 Madrid, Spain

**Keywords:** fibromyalgia, cognitive, execution, network analysis, fibro fog

## Abstract

The aim of this study was to quantify the multivariate relationships between clinical, cognitive performance, executive functioning, and psychological outcomes in women with fibromyalgia (FMS) using network analyses. Demographic (age, height, weight), clinical (pain history, pain intensity, and related disability), neurocognitive (D2 Attention test, Rey-Osterrieth Complex Figure for visual perception, “Digits D/R/I” tests of the WAIS-IV battery for working memory, the 5-Digit Test for mental inhibition, the Symbol Search for processing speed and the Zoo Test for planning/decision making) and psychological (depressive symptoms, anxiety levels, sleep quality, pain hypervigilance) variables were collected in 129 women with FMS and 111 healthy women. Network analyses were conducted separately for each group to quantify the adjusted correlations between the modeled variables and to assess their centrality indices (i.e., connectivity with other symptoms in the network and their importance in the network). The network identified 74 associations in FMS women and 46 associations in controls with small differences. The strongest correlations in both groups were found between different attention variables: d2_CON with d2_C, d2_O with d_2TR, and d2_CON with d2_TA. The most central variables were d2_TA, d2_C, and d2_CON (highest strength centrality in both groups) and anxiety levels and pain hypervigilance (highest harmonic centrality in FMS women). The strength centrality of the network was stable for women with FMS (CScor0.7: 0.68) but not for healthy women (CScor0.7: 0.28). This study found that attention variables are most relevant within a neurocognitive network and that psychological variables are most important for the treatment of women with FMS. The clinical implications of the current findings, such as the development of treatments targeting these variables, are discussed.

## 1. Introduction

Fibromyalgia syndrome (FMS) is a complex pain condition showing a prevalence of 6% of the worldwide population [1]. Patients with FMS can exhibit different physical (i.e., pain, fatigue, muscle weakness) and psychological (i.e., anxiety and depression, sleep disturbances) symptomatology [2]. The presence of these symptoms leads to a worse health-related quality of life [3]. Further, FMS represents a substantial economic burden to health care systems and society. It has been estimated that annual direct costs per patient with this condition range from USD 1250 to USD 8504 in Europe and from USD 1750 to USD 35,920 in the United States of America (USA) [4]. In addition, up to 60% of patients with FMS also report a third group of symptoms, that is, cognitive alterations such as memory loss, attention problems, or executive function deficits [5]. Evidence suggests that cognitive impairments seem to be specific since not all domains show the same affectation. For instance, learning memory and attention/psychomotor speed are two domains that are more affected (large effect size) than executive function and working memory domains (medium effect size) when comparing women with FMS to healthy women [6].

Previous studies investigating the association between different symptomatology (e.g., pain, function, disability, mood disorders, or cognitive aspects) in FMS have used statistical Pearson Product-Moment correlations or linear regressions. For instance, Larsson et al. observed that cognitive aspects, such as fear avoidance, were associated with physical aspects, such as muscular strength, in women with FMS [7]. Similarly, Devrimsel et al. found that grip force (a physical variable) was associated with related disability in a small sample of women with FMS [8]. The statistical analyses used in these previous studies ignore the potential for pairwise associations to arise from their interaction with another variable or the possibility of bidirectional relationships between variables [9].

Kumbhare and Tesio proposed a theoretical framework where reciprocal interactions between biology and cognitive behaviors are mutually integrated into patients with FMS [10]. Due to the heterogeneous cognitive symptoms that can be present in these individuals, FMS may emerge and be sustained by a collection of reciprocal interactions between clinical, psychological, cognitive, and physical systems. Accordingly, the use of traditional analyses, such as linear regressions, would be limited. Network analyses provide a methodology to understand complex relationships, addressing the aforementioned limitations of more traditional analyses [11]. More importantly, this type of analysis is able to identify the most important variables in an identified network, which could be used to potentially design therapeutic interventions [12].

A network approach showing complex interactions between altered nociceptive pain processing and emotional, psychological, and cognitive features has been previously applied in a sample of women with FMS [13]. This network identified that the altered nociceptive processing (i.e., PPT over the tibialis anterior), muscular strength (i.e., hand grip force), and physical function (i.e., Time Up and Go test) were those variables more relevant for the potential treatment of these patients [13]. However, this network did not include either neurocognitive variables or executive functions in their analysis. Expanding the understanding of relationships between cognitive impairments and executive function with psychological-associated factors in FMS patients is relevant for research and clinical practice. 

Accordingly, the current study applied, for the first time, network analysis to better understand the interactions between cognitive performance, executive function activities, and psychological and cognitive variables in women with FMS. The aims of this study were to (1) describe a network focusing on cognitive performance and executive function activities in a sample of women with FMS; (2) compare the network on cognitive performance and executive function activities between women with and without FMS; and (3) illustrate the potential of a network perspective for generating research questions and improving treatment strategies for patients with FMS.

## 2. Methods

### 2.1. Participants

A group of women with FMS was voluntarily recruited from various fibromyalgia associations in Madrid, Spain. Participants were required to be between 18 and 75 years old and have a confirmed FMS diagnosis from their rheumatologist [14]. Additionally, a group of women with no chronic pain history was recruited through local advertisements on social media platforms (Facebook, WhatsApp, Twitter) and notice boards at the Health Sciences Faculty of Universidad Rey Juan Carlos in Madrid. Both groups were excluded if they had (1) a history of whiplash; (2) undergone previous surgery; (3) co-existing medical conditions (e.g., rheumatoid arthritis); (4) neuropathic pain (e.g., radiculopathy); (5) current psychiatric diagnoses as per DSM-V [15] (e.g., major or mild neurocognitive disorders, schizophrenia); or (6) taking medications (e.g., antipsychotics, anticonvulsants, anticholinergics) that could impact cognitive functions [16]. The use of non-steroidal anti-inflammatory drugs (NSAIDs) in the group of women with FMS was permitted. 

The study design received approval from the Ethics Committee of Universidad Rey Juan Carlos (URJC 2508202218222). All information about this study was provided to the participants. Thus, all participants provided their written informed consent before participating. All the included variables were collected in a single individual session lasting 90 min. conducted by an experienced clinical neuropsychologist. Table 1 summarizes all neurocognitive areas evaluated.

### 2.2. Selection Attention

Selective attention and concentration were assessed using the Spanish version of the D2 Attention test—D2 [17]. Selective attention is described as “the capacity to focus on relevant aspects of a task while ignoring irrelevant ones, and to do so swiftly and accurately” [18]. The D2 test comprises 14 lines, each containing 47 characters (total items, 658). It features the letters “d” and “p” with possible dashes above or below. Participants must scan each line from left to right, marking each “d” with two dashes (either both above, both below, one above/one below) as relevant elements while ignoring other combinations. Each line is tested for 20 s, with the total test duration typically ranging from 8 to 10 min. The D2 tests have shown good internal consistency, validity, and reliability (r > 0.90) for measuring visual scanning accuracy and speed [19].

The following scores were obtained from the D2 test: d2_TR, the number of elements tried on the 14 lines; d2_TA, the number of correct relevant elements identified; d2_O, the number of relevant elements tried but not properly marked (omitted elements); d2_C, the number of irrelevant elements marked (commissions); d2_TOT, total test effectiveness calculated as TR − (O + C); d2_CON, concentration index calculated as TA-C; d2_TR+, line with a greater number of tried elements; d2_TR−, line with a lower number of elements tried; and d2_VAR, variation index or difference calculated as TR+ (-) TR−.

### 2.3. Visuospatial Memory

Visual perception, visuo-constructional ability, and spontaneous memory retention were evaluated with the Rey-Osterrieth Complex Figure (ROCF). This test assesses the retention of visual details, the ability to organize and integrate parts of a figure, and mental manipulation of the figure [20]. Participants first copy a geometric figure (comprising 18 black lines) onto a piece of paper. Then, the participants are asked to draw the figure from memory again immediately afterward (immediate recall) and after 20–30 min (delayed recall) without the sheet. No instructions are provided to memorize the figure since the task tries to measure what is spontaneously kept in mind. The following scores were obtained: ROCF_Copy, immediate recall and delayed recall points (calculated copy points/maximum points); ROCF_Recall, recall percentage (delayed recall points/immediate recall points); and ROCF_TimeCopy, the time needed for doing the copy. The ROCF has also shown good psychometric properties [21], including excellent intra- and inter-rater reliability [22].

### 2.4. Executive Function

Working memory was measured using the “Digits D/R/I” subtest from the Wechsler Adult Intelligence Scale WAIS-IV [23]. This subtest includes three tasks: digit span forward (DSF, repeating digits in the same order as presented), digit span backward (DSB, repeating digits in reverse order), and digit span sequencing (DSS, repeating numbers in ascending order).

Mental inhibition was assessed using the “response inhibition index” from the 5-Digit Test-FDT [24], a STROOP-like task. This test comprises the following four sections: Reading, Counting, Election, and Alternation, each with 50 items. The Reading and Counting sections assess automatic and simple processes, while Election and Alternation evaluate more complex processes. Scores are derived from counting errors in each section and multiplying by the time taken and include Decoding_FDT (time needed to read all numeric items), Retrieving_FDT (time needed to read non-numeric items such as asterisks), Inhibiting_FDT (time needed to read identical numeric items), and Shifting_FDT (time needed to read identical numeric items interspersed with other numeric items). The reliability of the FDT has been shown to be moderate-to-excellent (ICC from 0.59 to 0.97) [25]. 

Processing speed was tested with the “Symbol Search” (SS) subtest from the Wechsler Adult Intelligence Scale (WAIS-IV) [26]. This paper-and-pencil test includes a key area with nine pairs of digits and symbols first and a second response area with random digits and blanks. Participants must fill in the blanks with symbols as fast as possible within 120 s. The SS has shown good test-retest reliability (r from 0.70 to 0.80) [27]

Planning/decision-making was evaluated using the Zoo Map Test [28]. It measures organizational skills, planning, and problem-solving to achieve a specific goal and includes two versions: 1, the version to evaluate planning ability in an environment where no pattern should be followed, and everything depends on the person; 2, the version evaluating the use of a concrete strategy of the external type. Scores are calculated by subtracting the number of errors from the sequence score, with the total error score ranging from 0 to 16 points. A score between 11 and 16 is considered normal, while a score of 10 or below indicates some degree of deficiency.

### 2.5. Clinical, Psychological, and Cognitive Variables

A 10-point (0: no pain, 10: maximum pain) Numerical Pain Rate Scale (NPRS) was used to evaluate the mean pain intensity [29]. The NPRS has shown good concurrent validity (r = 0.96, 95% CI 0.92–0.97) in individuals with FMS [30].

The Spanish version of the Fibromyalgia Impact Questionnaire (FIQ) was used to evaluate function and disability due to FMS [31]. The FIC provides a score ranging from 0 to 100 points, with a higher value means more negative impact of the disease on function [32]. The Spanish version of the FIQ has shown internal consistency and reliability (r > 0.86) [33].

The Spanish version of the Hospital Anxiety and Depression Scale (HADS) was used for assessing the presence of anxiety (HADS-A, seven items) and depressive (HADS-D, seven items) symptoms [34]. Each item ranges from 0 to 3, and the total score of each scale ranges from 0 to 21 points. A score of ≥8 points suggests the presence of anxiety or depressive symptoms with good sensitivity and specificity [35]. The psychometric properties of the HADS have been shown to be good in the general population [34]. In FMS, the HADS has also shown good validity and reliability (r ranging from 0.83 to 0–87) [36].

The Spanish version of the Pittsburgh Sleep Quality Index (PSQI) was used to assess the quality of sleep [37]. The PSQI provides an overall score ranging from 0 to 21 points, where a cut-off of ≥8 points indicates poor sleep quality. The Spanish version of the PSQI has shown good internal consistency and acceptable test-retest reliability (r = 0.773) in women with FMS [37].

Pain hypervigilance was finally assessed with the Spanish version of the short-form nine-items Pain Vigilance and Awareness Questionnaire (PVAQ-9, 0 to 45 points), which has shown good reliability (r = 0.82) and proper convergent/divergent validity in FMS [38].

### 2.6. Statistical Analysis

Patients with FMS and controls were separately analyzed using the R software v.4.1.1 (RStudio, Boston, MA, USA) for Windows 10 and specific libraries for network estimation and stability analysis (data.table, dplyr, ggplot2, gridExtra, kableExtra, plotly, igraph, ggpubr, tidyr, arsenal, bootnet, pander, xtable, DT, summarytools, qgraph, initr, huge, missForest, RColorBrewer, mgm, CINNA and scales).

Firstly, an exploratory data analysis was conducted to detect missing values in the dataset. One patient (0.77%) was dropped from the analysis as the pain intensity value was missing. No missing data were identified in the control group. Then, descriptive statistics (mean, standard deviation, interquartile range, and coefficient of variation for continuous variables and frequency and percentage for categorical variables) and histograms were calculated for both samples separately. 

Network analyses were conducted according to previous studies [39,40]. Thus, the graph within the FMS group was built based on 29 nodes as continuous variables (age, height, weight, time with pain, ROCF_Copy, ROCF_Recall, ROCF_Time Copy, d2_TR, d2_TA, d2_O, d2_C, d2_TOT, d2_CON, d2_VAR, Symbol Search, Digits_SF, Digits_SB, Digits_SS, Decoding_FDT, Retrieving_ FDT, Inhibiting_FDT, Shifting_FDT, Zoo Map Test, pain intensity, HADS-A, HADS-D, PVAQ-9, PSQI, and FIQ) for the patient group. 

For the control group, the same variables were included except those related to pain symptoms, leading to a network with 26 nodes as continuous variables.

The association (if it exists) between the nodes is illustrated with lines. Thicker lines represented strong association, while thinner lines represented weak association. Positive correlations are represented by a green line, while negative correlations are represented by a red line. Strength centrality (a blunt measure indicating the total level of involvement of a node in the network, being a clinically useful indicator to determine which outcome could induce direct changes in other variables), closeness centrality (the inverse sum of the distances of shortest paths between the node with other nodes to identify the outcome that could induce changes to other outcomes quicker than other peripheral outcomes), and harmonic centrality (instead of betweenness as some nodes were not connected, it was calculated to calculate the average distances of a node with the rest) indices were calculated [41].

Finally, the stability of the model was analyzed. Upon conducting 2000 iterations, the variability in edge weights and centrality indices was examined to determine 95% confidence intervals. Broad confidence intervals can make deciphering the edge strength challenging, but this does not affect its existence, as LASSO has already executed model selection. To gauge changes in the centrality indices (CS-coefficient), a bootstrap method that involves dropping a portion of subjects was employed [12]. This technique recalculates the network and compares three centrality measures. The CS-coefficient, or correlation stability, showcases the maximum amount of data that can be omitted (ideally more than 0.25) while maintaining a correlation above 0.7 with the primary centrality indices (with 95% certainty).

## 3. Results

From a total of 150 women with FMS screened for eligibility, 20 (13.3%) were excluded: older than 75 years (n = 6), presence of neurocognitive disorder (n = 5), regular intake of anticholinergics (n = 4), refused to participate (n = 3), and no medical diagnosis of FMS (n = 2). Finally, 130 women with FMS (mean age: 54.7, SD: 9.4) fulfilled all inclusion criteria and were accepted to participate, but due to missing data on pain intensity in one, the final sample of women with FMS was 129. From a total of 125 asymptomatic women who responded to the announcements, 111 women (mean age: 55.3; SD: 13.9) served as controls. The reasons for exclusion were refusal to participate (n = 6), neurocognitive disorder (n = 4), taking opioid medication for hernia disc (n = 2), and being older than 75 years old (n = 2). Descriptive statistics of either group can be found in Table 2. 

**Figure 1:**
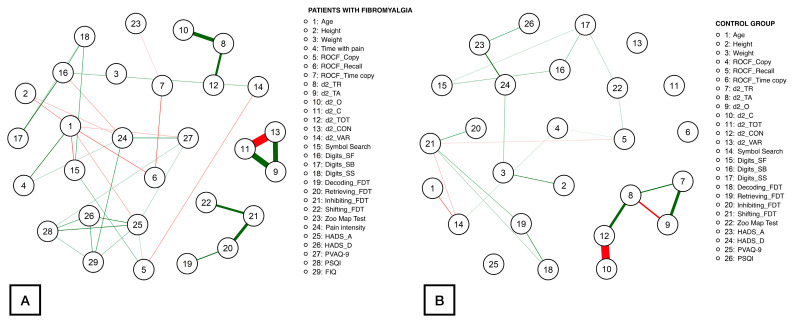
Network analysis of the association between demographic, clinical, attention aspects, executive function, cognitive, and psychological measures in women with fibromyalgia (**A**) and healthy controls (**B**). Notes: Edges represent connections between two nodes and are interpreted as the existence of an association between two nodes, adjusted for all other nodes. Each edge in the network represents either positive regularized adjusted associations (green edges) or negative regularized adjusted associations (red edges). The thickness and color saturation of an edge denotes its weight (the strength of the association between two nodes).

Figure 1 illustrates the network obtained for patients (Figure 1A) and controls (Figure 1B). Up to 74 correlations were identified within the patient network, being the correlations between d2_CON with d2_C (*ρ* = −1.39), d2_C with d2_TA (*ρ* = 1.03), d2_CON with d2_TA (*ρ* = 0.92), d2_O with d2_TR (*ρ* = 0.74), Inhibiting_FDT with Retrieving_FDT (*ρ* = 0.63), and d2_TOT with d2_TR (*ρ* = 0.54) the strongest. The rest of the correlation values ranged from |0.05| to |0.52|. Fewer correlations were found within the healthy controls network (46 correlations). In this case, the most notable correlations were observed between d2_CON with d2_C (*ρ* = −1.61), d2_O with d_2TR (*ρ* = 0.83), d2_CON with d2_TA (*ρ* = 0.79), d2_O with d2_TA (*ρ* = −0.63), and d2_TA with d2_TR (*ρ* = 0.55). The absolute correlation coefficients for the remaining correlations ranged from |0.05| to |0.48|.

**Figure 2:**
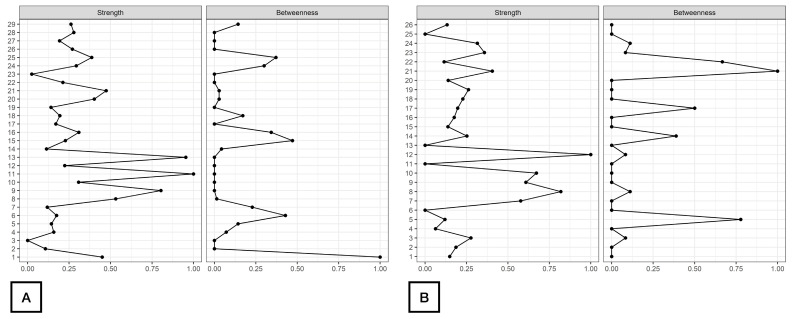
Centrality measures of Strength and Betweenness of each node in the network in women with fibromyalgia (**A**) and healthy controls (**B**). Note: Centrality value of 1 indicates maximal importance, and 0 indicates no importance.

**Figure 3:**
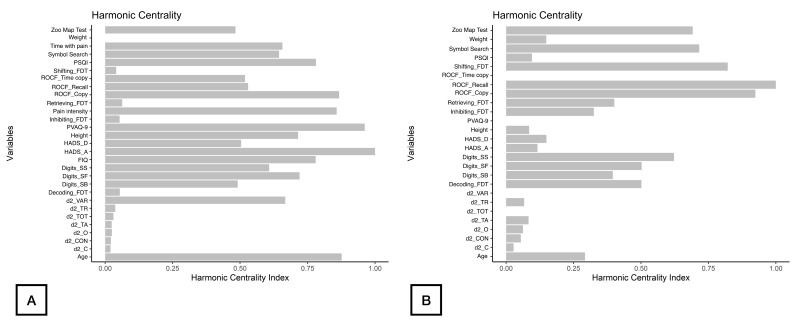
Harmonic Centrality measure of each node in the network in women with fibromyalgia (**A**) and healthy controls (**B**). Note: Centrality value of 1 indicates maximal importance, and 0 indicates no importance.

As displayed in Figure 2, d2_TA, d2_C, and d2_CON were the nodes with the highest strength centrality in both patients (nodes 9, 11, 13, Figure 2A) and controls (nodes 8, 10, 12, Figure 2B). Age (node 1), ROCF_Recall (node 6) and Symbol_Search (node 15) were the nodes showing the highest betweenness centrality in patients with FMS (Figure 2A), whereas ROCF_Recall (node 5) and Shifting_FDT (node 21) were those with the highest betweenness centrality in healthy controls (Figure 2B). Figure 3 reveals that clinical variables, e.g., HADS-A (node 25) and PVAQ-9 (node 27), as well as ROCF_Copy (node 5), were the nodes showing the highest harmonic centrality in patients with FMS (Figure 3A), whereas ROCF_Recall (node 5), ROCF_Copy (node 4) and Shifting_FDT (node 21) were the nodes with greatest harmonic centrality in controls (Figure 3B).

**Figure 4:**
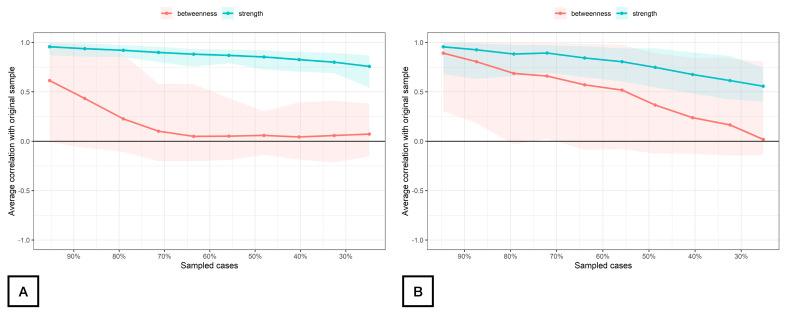
Average correlations between centrality indices of networks sampled with persons dropped and networks built on the entire input dataset at all follow-up time points in women with fibromyalgia (**A**) and healthy controls (**B**). Note: Lines indicate the means, and areas indicate the range from the 2.5th quantile to the 97.5th quantile.

The stability of the modeled network is displayed in Figure 4. The betweenness measures of the network (CScor = 0.7) were unstable at 0.00 for both women with FMS (Figure 4A) and controls (Figure 4B), whereas the strength centrality measure was found stable at CScor = 0.7 for patients with FMS (0.68, Figure 4A) and poorly stable for controls (0.28, Figure 4B) according to the cut-off scores described in the literature [42]. The closeness centrality measure could not be assessed with bootstrapping since there were unconnected nodes.

## 4. Discussion

The use of network analysis in women with FMS revealed that attention variables had the highest correlation within the neurocognitive network and that psychological variables had the greatest significance for the change in the other variables of the network in women with FMS. The same analysis conducted in a cohort of healthy women showed that attention variables had the highest correlation within the neurocognitive network; however, the psychological construct was not relevant in the control group.

This is the first study describing a network of neurocognitive and psychological variables and executive functions in women with FMS. The network identified revealed that attention variables evaluated with the D2 attention test (particularly d2_TA, d2_C, or d2_CON) were those most inter-correlated and showed the highest strength centrality. Attention has been a key domain when working with patients with FMS [43,44]. In fact, the relevance of the attention process can be doubled in FMS: 1, patients with FMS have more attention deficits than pain-free controls [6]; and 2, patients with FMS engage in descending pain modulatory system if attentional tasks are appropriately stimulated [45]. A possible hypothesis would be that sensitization processes involved in FMS pathogenesis could contribute to attention deficits [46]. Thus, there is evidence that increased activity in the pain neuromatrix amplifies hyperalgesia and allodynia, which characterize FMS [47]. This supports the theory that higher sensitization could be associated with deeper attentional alterations in these patients [48]. In fact, brainstem networks involved in pain processing and attention partially overlap since the medial and lateral prefrontal areas, as well as the anterior cingulate, participate in both nociception and attention processing [49]. Therefore, the presence of altered nociceptive processing takes greater demands on these brainstem areas and, therefore, could reduce attention processing resources [50,51]. Nevertheless, the relevance of attention variables in the identified network does not clearly support the clinical relevance of this construct. In fact, treatment of attention deficits, e.g., by using attention bias modification interventions, did not produce substantive improvements in pain, disability, or psychological constructs in patients with FMS [52].

The executive function showing the highest correlation in the identified network was mental inhibition, as assessed with the FDT; however, it should be noted that the current literature about deficits in mental inhibition in FMS is not conclusive [53]. For instance, Wu et al. did not find deficits in mental inhibition [6], whereas Bell et al. observed moderate-large effect sizes for this deficit in inhibitory control [54] between patients with and without FMS. These results support the idea that impairments in executive function are specific to domains. A deficit in mental inhibition could be related to a hypoactivation in the premotor cortex (PMC), the supplementary motor area (SMA), the medial cingulate cortex (MCC) and the putamen in individuals with chronic pain [55]. Thus, there is substantial overlap between the neural brainstem networks involved in inhibition and pain perception, which is in agreement with the hypothesis that excessive attentional focus on a threat (pain) can alter the activation of inhibitory systems [55]. In fact, the term “inhibition network” is of higher interest since it seems that the brainstem is organized into different networks [56]. The relevance of inhibition is further supported by the fact that a lack of inhibition has been associated with lower pain tolerance and greater attention to it (hypervigilance) in pain-free subjects [57]. Therefore, inhibition seems to be integrated into a network with pain interference and cognitive functioning. 

The decision-making process for treating patients with FMS can be challenging for clinicians since these patients suffer from a plethora of overlapping symptoms, making it difficult to determine which symptom or construct should be treated first. An important finding from this study was that two psychological-cognitive aspects, i.e., anxiety and hypervigilance to pain, showed the highest harmonic centrality, suggesting that these variables may be relevant for inducing changes within the network. This means that clinical management of anxiety levels and pain hypervigilance can be crucial for treating FMS patients. Current evidence supports that treatment strategies targeting physical aspects, e.g., exercise, also have a positive impact on psychological aspects such as anxiety/depressive levels in FMS patients [58]. However, it should be noted that psychological/cognitive interventions can be effective in improving anxiety and depressive levels but not physical symptoms such as fatigue [59]. Accordingly, management of anxiety levels should be multimodal in FMS. 

As expected, the addition of neurocognitive approaches, such as pain neuroscience education, increases the positive effect of these interventions [60]. In fact, pain neuroscience education reduces mal-adaptative cognitive behaviors such as hypervigilance to pain and kinesiophobia levels. The current literature indicates that pain hypervigilance in FMS patients is the result of a dynamic process when the individual anticipates a potential threat, e.g., pain, and promotes an exaggerated response against this threat, thus intensifying and/or promoting more symptomatology [61]. Thus, this mal-adaptative behavior can also promote stress and anxiety in patients with FMS, perpetuating a vicious cycle of pain and related disability [62]. Cognitive behavior approaches targeting pain hypervigilance by managing how to cope with pain have been demonstrated to be effective in FMS, although evidence is still limited [63].

Finally, long-term visual memory, as assessed with ROC, also showed betweenness centrality (ROCF_Recall) and harmonic centrality (ROCF_Copy) in women with FMS. Preliminary evidence suggests that memory deficits in FMS can be challenging for people with FMS when faced with decisions related to previous events or situations. Additionally, this situation could lead to feelings of low capacity or ability against daily life situations and lead to stress and anxiety levels [59]. In fact, memory deficits have several implications for patient engagement and retention of information provided in therapy [64], an aspect highly relevant for cognitive behavior interventions. In such a scenario, changes in gray and white matter volume of the hippocampus [65], a structure particularly related to memory, could play a central role in various phenomena associated with FMS, such as lower levels of glutamate [66] or N-acetylaspartate [67]. In fact, long-term deficits of some glucocorticoids (cortisol) in the hippocampus are associated with metabolic abnormalities that would lead to a loss of functionality and neuronal death, producing morphological brain changes and being behind the memory difficulties, as seen in patients with FMS [68].

The results of this study should be considered according to its strengths but also potential limitations. The main strengths are the use of network analysis for the first time in this population and also the inclusion of a cohort of asymptomatic individuals to see if the identified network is also valid in healthy controls. However, it should be noted that we only included women with FMS; accordingly, the identified network should not be applied to men with FMS. Second, although we excluded patients actively taking psychoactive drugs or other medications that may affect cognitive function, a potential long-lasting effect of medications that patients have taken in the past on cognitive performance cannot be totally ignored. In fact, future studies could group patients according to the type of medication intake for the identification of their influence. Finally, we included a battery of neurocognitive tests assessing specific domains of cognition in FMS. As a result, further studies using a wider battery of neuropsychological tests capable of covering all of the components of executive functions and attentional and memory processes are needed to identify more expanded networks in this population.

## 5. Conclusions

The use of network analysis in women with FMS revealed that attention variables showed the highest correlation within the neurocognitive network and that psychological variables were the most relevant for modifying the remaining variables of the network. Thus, attention variables also showed the highest correlation within the neurocognitive network in a cohort of asymptomatic women; however, the psychological construct was not relevant in the control group. This study illustrates the potential of a network perspective for generating research questions and improving treatment strategies for FMS patients.

## Figures and Tables

**Table 1:** Cognitive domains assessed and neuropsychological tasks.

Cognitive Domains	Neuropsychological Tests	Outcomes	Administration
Short-term and Working Verbal Memory	Digit Span Forward	Span of digits	Auditory - Oral
	Digit Span Backward	Auditory Working Memory	
	Digit Span Sequencing	Auditory Working Memory	
Selective Attention	D2 Test of Attention	D2_TR	Visual - Manual (Paper)
		D2_TA	
		D2_TOT	
		D2_CON	
		D2_VAR	
		D2_O	
		D2_C	
Long-Term Visual Memory	ROCF	ROCF_Copy	Visual - Manual (Paper)
		ROCF_Recall	
		ROCF_TimeCopy	
Processing Speed	Symbol Search	Symbol Search direct punctuation	Visual - Manual (Paper)
Mental Inhibition and Flexibility	Five Digits Test FDT	Decoding_FDT	Visual - Oral
		Retrieving_FDT	
		Inhibiting_FDT	
		Shifting_FDT	
Planning	Zoo Map Test (BADS)	Personal test punctuation	Visual - Manual (Paper)

BADS: Behavioral Assessment of the Dysexecutive Syndrome; D2_TR = total number of items answered; D2_TA = number of items answered correctly; D2_O = errors of omission committed; D2_C = commission errors made; D2_TOT = number of elements processed minus the total number of errors committed; D2_CON = number of relevant elements marked minus the number of commissions; D2_VAR = variation index d2; ROCF: Rey-Osterrieth Complex Figure test; ROCF_Copy = direct scoring in the copy phase of the Rey-Osterrieth Complex Figure; ROCF_Recall = direct scoring in the delayed Recall phase of the Rey-Osterrieth Complex Figure; Symbol Search = direct scoring of correctly answered items; Decoding_FDT = time in seconds to read all numeric-items; Retrieving_FDT = time in seconds to read all non-numeric ítems; Inhibiting_FDT = time in seconds to read numeric items; Shifting_FDT = time in seconds to read non-numeric items; Zoo Map Test = direct score in carrying out planning test.

**Table 2:** Clinical and neurocognitive data of women with and without FMS.

	Controls (n = 111)	Patients (n = 129)
Age (years)	Mean (SD) 55.3 (13.9)	Mean (SD) 54.7 (9.4)
Height (cm)	Mean (SD) 161.0 (6.9	Mean (SD) 162.3 (7.1)
Weight (kg)	Mean (SD) 65.7 (12.0)	Mean (SD) 72.7 (15.1)
Time with pain (years)	-----	Mean (SD) 21.3 (13.0)
ROCF_Copy	Mean (SD) 32.2 (4.0)	Mean (SD) 30.9 (7.1)
ROCF_Recall	Mean (SD) 15.4 (7.0)	Mean (SD) 12.5 (7.1)
ROCF_Time Copy	Mean (SD) 3.9 (14.8)	Mean (SD) 3.1 (1.0)
d2_TR	Mean (SD) 383.8 (91.3)	Mean (SD) 332.1 (90.4)
d2_TA	Mean (SD) 134.3 (41.4)	Mean (SD) 107.6 (40.1)
d2_O	Mean (SD) 28.2 (29.9)	Mean (SD) 32.8 (38.1)
d2_C	Mean (SD) 3.8 (10.6)	Mean (SD) 4.9 (13.0)
d2_TOT	Mean (SD) 350.3 (93.4)	Mean (SD) 290.4 (87.9)
d2_CON	Mean (SD) 131.2 (45.5)	Mean (SD) 103.7 (44.0)
d2_VAR	Mean (SD) 14.8 (7.0)	Mean (SD) 15.5 (7.4)
Symbol_Search	Mean (SD) 31.0 (8.7)	Mean (SD) 26.3 (8.5)
Digits_SF	Mean (SD) 8.3 (2.0)	Mean (SD) 7.6 (2.0)
Digits_SB	Mean (SD) 7.5 (2.0)	Mean (SD) 6.6 (1.8)
Digits_SS	Mean (SD) 7.9 (2.4)	Mean (SD) 6.8 (2.1)
Decoding_FDT	Mean (SD) 19.9 (3.7)	Mean (SD) 25.6 (9.7)
Retrieving_FDT	Mean (SD) 22.3 (4.4)	Mean (SD) 29.6 (9.7)
Inhibiting_FDT	Mean (SD) 36.3 (10.3)	Mean (SD) 48.4 (25.7)
Shifting_FDT	Mean (SD) 49 (14.9)	Mean (SD) 62.0 (33.9)
Zoo Map Test	Mean (SD) 12.2 (3.4)	Mean (SD) 10.9 (4.1)
NPRS (0–10)	-----	Mean (SD) 7.6 (1.3)
HADS-A (0–21)	Mean (SD) 5.9 (4.1)	Mean (SD) 13.5 (3.9)
HADS-D (0–21)	Mean (SD) 3.5 (3.6)	Mean (SD) 11.05 (4.3)
PVAQ-9 (0–45)	Mean (SD) 16.35 (12.6)	Mean (SD) 29.4 (7.8)
PSQI (0–21)	Mean (SD) 7.2 (4.1)	Mean (SD) 15.2 (3.8)
FIQ (0–100)	----	Mean (SD) 75.2 (12.2)

SD: Standard Deviation; ROCF_Copy = direct scoring in the copy phase of the Rey-Osterrieth Complex Figure; ROCF_Recall = direct scoring in the delayed Recall phase of the Rey-Osterrieth Complex Figure; d2_TR = total number of items answered; d2_TA = number of items answered correctly; d2_O = errors of omission committed; d2_C = commission errors made; d2_TOT = number of elements processed minus the total number of errors committed; d2_CON = number of relevant elements marked minus the number of commissions; d2_VAR = variation index d2; Symbol Search = direct scoring of correctly answered items; Digits_SF = Digit Span Forward; Digits_SB = Digit Span Backward; Digits_SS = Digit Span Sequencing; Decoding_FDT = time in seconds to read all numeric-items; Retrieving_FDT = time in seconds to read all non-numeric items; Inhibiting_FDT = time in seconds to read the group of each numeric item; Shifting_FDT = time in seconds to read the group of the same numeric item mixed with numeric-items within a box; Zoo Map Test = direct score in carrying out the planning test. NPRS: Numerical Pain Rating Scale; HADS: Hospital Anxiety and Depression Scale (A: anxiety, D: depression); PVAQ-9: Pain Vigilance and Awareness Questionnaire; PSQI: Pittsburg Sleep Quality Index; FIQ: Fibromyalgia Impact Questionnaire.

## Data Availability

Materials and analysis code for this study are not available in any repository; however, we will make our data accessible upon request to the corresponding author.
